# Underinvestment in nutrition research for at‐risk populations: An analysis of research funding awarded in Australia from 2014 to 2021

**DOI:** 10.1111/1747-0080.12740

**Published:** 2022-05-03

**Authors:** Laura Alston, Rebecca Raeside, Si Si Jia, Stephanie R. Partridge

**Affiliations:** ^1^ The Global Obesity Centre, Institute for Health Transformation Deakin University Geelong Victoria Australia; ^2^ Deakin Rural Health, School of Medicine, Faculty of Health Deakin University Warrnambool Victoria Australia; ^3^ Colac Area Health Colac Victoria Australia; ^4^ Engagement and Co‐design Hub, School of Health Sciences, Faculty of Medicine and Health The University of Sydney Sydney New South Wales Australia; ^5^ Prevention Research Collaboration, Charles Perkins Centre, Sydney School Public Health The University of Sydney Sydney New South Wales Australia

**Keywords:** Aboriginal and Torres Strait Islander, diet, food and nutrition, funding, health services research, rural health, socioeconomically disadvantaged

## Abstract

**Aim:**

To determine the proportion of research projects funded by the National Health and Medical Research Council and Australian Research Council research funding from 2014 to 2021 that aimed to understand or improve dietary behaviours for at‐risk populations in Australia and estimate the proportion of total funding allocated during this period.

**Methods:**

Retrospective analysis of the publicly available National Health and Medical Research Council and Australian Research Council funding grants over the 8 years from 2014 to 2021 (*n* = 18 098). At‐risk dietary populations included people living in rural and remote Australia, Aboriginal and Torres Strait Islander people, or people living in socioeconomically disadvantaged areas. Descriptive analysis was undertaken.

**Results:**

In total, 144 out of 18 098 (0.8%) individual grants totalling $96.8 million were identified relating to nutrition research from 2014 to 2021. Out of the 144, only 21 ($19.6 million; 0.1%) of all National Health and Medical Research Council grants were identified for nutritionally at‐risk populations, with the majority focused on Aboriginal and Torres Strait Islander people (15/21). The National Health and Medical Research Council and Australian Research Council grants that aimed to improve human dietary behaviours increased by 0.66% and 0.58%, respectively, from 2014 to 2021. However, the National Health and Medical Research Council grants aiming to improve nutritional behaviours in at‐risk populations decreased by 0.04% over the 8 years.

**Conclusions:**

Despite slight increases in the proportions of funding to improve dietary behaviours over the past decade, nutrition research specifically targeting at‐risk groups is scarce and appears to have decreased over time. Insufficient investment in research for these groups presents a risk for widening health disparities now and into the future. As such, they must be further supported and considered in the design of future funding schemes.

## 1 INTRODUCTION

Globally in 2017, 11 million deaths and 255 million disability‐adjusted life years were attributable to dietary risk factors.^1^ In Australia, in 2018, dietary risk factors represented 5.4% of the total burden of disease.^2^ Dietary risks were the third leading risk factor contributing to disease burden after tobacco use and overweight and obesity around the world.^1^ Due to the many social and commercial determinants of health impacting dietary intake,^3^Australians tend to have poor nutritional behaviours and this is consistent across all age groups. Some subgroups, particularly those of low socioeconomic status or those living outside of major cities, are less likely to meet dietary guidelines^4^ due to inequitable access to healthy and affordable foods, which marginalises them.^3^ Very little progress in improving population diets has been observed in the Australian population over time.^1^, ^5^, ^6^ The 2020 Global Nutrition Report highlights the historical underinvestment in efforts to improve nutrition across the globe as a contributing factor to increasing preventable health burden.^7^ The report outlines the risks of ongoing health inequities if the lack of investment continues and the need to emphasise nutritional wellbeing for all, particularly the most high‐risk populations. Addressing health inequities is especially important in planning for post‐COVID‐19 recovery worldwide.^7^

There is significant potential to improve population health by optimising dietary intake and reducing preventable morbidity and mortality.^2^, ^5^ For example, fruit and vegetable intakes are well documented to reduce the risk of non‐communicable diseases. Multiple randomised controlled trials and prospective cohort studies have shown that adequate consumption of fruits and vegetables positively affects serum lipid levels,^8^ blood pressure,^9^ insulin resistance,^10^ makers of inflammation,^11^ in turn reducing risks from non‐communicable diseases like cardiovascular diseases, diabetes, chronic kidney disease, some cancers and total mortality.^12^ Research and interventions to improve dietary intake across all subpopulations and age groups are urgently needed to improve overall health.

Theoretical changes in diet, for one of Australia's most significant contributors to mortality (cardiovascular disease), have shown great promise in reducing the risk of mortality and morbidity, which leads to less burden on the health system.^13^ Significant gains can be made from small changes in diet, with studies observing a reduction in risk per gram increase in intake of fruit and vegetables. For example, in a pooled sample of 937 655 participants, a dose–response relationship for coronary heart disease per 477 g/day for combined fruit and vegetable intake (risk ratio [RR] of 0.88) was observed, with decreasing risks beyond this amount.^14^ Despite the potential for reducing risks, national health survey data has shown that Australians generally do not meet dietary guidelines,^15^ with only 7.5% of adults consuming the recommended amounts for vegetable intake in 2018.^4^ Alston et al. modelled cardiovascular disease deaths in metropolitan and rural Australia and found that if everyone could meet public health recommendations for dietary intake and physical activity, 14 892 deaths would be potentially averted annually.^13^

To date, research into improving diet has shown much promise to reduce chronic disease.^16^, ^17^ However, with rapidly changing and unhealthy food environments,^18^, ^19^ positive dietary change is challenging, and it is a significant public health concern as detailed in the decadal plan for the science of nutrition.^20^ The decadal plan for nutrition is a plan, convened by the Australian Academy of Science, to better utilise and develop the scientific evidence to help address the double burden of malnutrition and obesity, which have been identified as the major nutrition challenges of our time. The plan also focuses on promoting a secure nutrition and food future across all subpopulations in Australia.^20^ Addressing dietary risks is also an international priority with the World Health Organization.^21^ Dietary intake, quality and diet‐related health outcomes follow the social gradient in Australia.^4^ These nutritionally ‘at‐risk’ groups include people living in rural and remote Australia, Aboriginal and Torres Strait Islander peoples,^22^ people with a disability, or people living in socioeconomically disadvantaged areas.^4^, ^23^ These populations face increased challenges in accessing and consuming affordable healthy foods and tend to have higher rates of overweight and obesity, diabetes, cardiovascular disease, and poor oral health.^2^, ^5^, ^20^, ^24^, ^25^ These groups are in particular need of equitable and low‐cost population health initiatives that have been evidenced informed and based on current diet–health relationships, that also address societal and commercial factors, as outlined in the decadal plan for the science of nutrition.^20^ Low investment in population health initiatives among at‐risk populations will hinder progress and undoubtedly increase the likelihood of these groups experiencing ongoing unequal health status for decades to come. It is essential that research funding schemes are reviewed on a regular basis to assess if funding distribution is applied relative to the need for at‐risk populations, to ensure progress is made in addressing health among population groups that experience unequal health. Nutrition research will play an important role in addressing current health gaps and in preventing the exacerbation of further inequity.

This study aimed to determine the proportion of research projects funded by the National Health and Medical Research Council (NHMRC) and Australian Research Council (ARC) in the period 2014–2021 that aimed to understand or improve dietary behaviours for at‐risk populations in Australia, and to estimate the proportion of total funding this represented in 2014–2021.

## 2 METHODS

This study is a retrospective analysis of the publicly available NHMRC and ARC data sets that were available online. Data sets were available over an 8‐year period from 2014 to 2021.^26^, ^27^ These data were non‐human data available on public websites and did not require ethical review by an ethics committee. Grants from NHMRC and ARC were selected as these funding bodies represent the two largest, nationally competitive peer‐reviewed funders for Australian research and development. Each data set was independently coded by three authors to determine the proportion of grants in the data set that aimed to understand or improve dietary behaviours, and deliver benefits to at‐risk populations. Populations at risk of inadequate nutritional intake were defined by existing literature. They included people living in rural and remote Australia, Aboriginal and Torres Strait Islander peoples, people living in socioeconomically disadvantaged areas, or people living with a disability.^4^, ^13^, ^22^, ^28^, ^29^ NHMRC targeted calls for research were included. All infrastructure or equipment and facilities support grants were excluded. Basic science and infrastructure grants were also excluded because the potential outcomes would benefit all Australians. Grants that focused only on policy analyses or assessments, mathematical modelling (or simulation type studies), supplements such as parental/enteral nutrition in critically ill patients were also excluded on the basis that outcomes do not directly target food‐related dietary behaviours. Another author independently cross‐checked all identified grants and resolved any discrepancies.

The search was conducted in two stages to (1) identify all grants that specifically aimed to understand or improve human dietary behaviours and (2) deliver benefits to at‐risk populations. First, for all NHMRC and ARC grants, the ‘Primary Field of Research’ and the ‘FoR Category’ were filtered, respectively, for ‘Nutrition and Dietetics’. All grant scientific titles and grant summaries/media summaries or plain descriptions were reviewed, and those that aimed to understand or improve human dietary behaviours were identified. To ensure a comprehensive search, a filter search strategy was conducted using the five research keywords or five health keywords for all NHMRC grants, and a keyword search was conducted for all ARC grants. The keyword search terms were broad, including truncations and synonyms of ‘nutrition’, ‘diet’, ‘food’, ‘food behaviours’, and ‘eating behaviours’. Second, the grants identified in Stage 1 were screened to determine whether they aimed to benefit at‐risk populations. All scientific titles and grant summaries/media summaries or plain descriptions, and research and health keywords were reviewed related to the at‐risk groups of interest (people living in rural and remote Australia, Aboriginal and Torres Strait Islander peoples, or people living in socioeconomically disadvantaged areas). Funding totals were aggregated for both stages and compared to the total funding for the period 2014–2021 using descriptive statistics. All data coding and analysis were conducted using Microsoft Excel (version 16.54, Microsoft 365).

## 3 RESULTS

Table 1 shows the total NHMRC and ARC grants from 2014 and 2021, along with the mean proportion of funding allocated across the time. In total, 144 out of 18 098 (0.8%) individual grants were identified relating to nutrition research from 2014 to 2021 (Table 1). Eighteen were identified from ARC and 126 from NHMRC schemes. Of the 144, only 21 (21/18098, 0.1%) individual grants were identified relating to nutrition research for at‐risk populations. All 21 individual grants were identified from NHMRC, and none were identified from ARC. Of the 21 grants, 15 were focused on Aboriginal and Torres Strait Islander peoples, 5 were focused on people living in socioeconomically disadvantaged areas, and 2 were focused on people living in rural and remote Australia. No grants were identified focusing on improving dietary behaviours among people living with a disability.

**TABLE 1:** Total Australian Research Council and National Health and Medical Research Council grants by year from 2014 to 2021 that aim to improve human dietary behaviours and among at‐risk populations

Funding scheme	2014			2015			2016			2017			2018			2019			2020			2021			Total 2014–2021		
	*n*	$	%	*n*	$	%	*n*	$	%	*n*	$	%	*n*	$	%	*n*	$	%	*n*	$	%	*n*	$	%	*n*	$	%
ARC																											
Nutrition focused	3	$571 606.00	0.05	1	$210 674.00	0.04	2	$749 120.00	0.1	4	$1 521 714.00	0.18	0	$0.00	0	3	$1 077 751.00	0.17	2	$923 147.00	0.09	3	$4 235 093.00	0.63	18	$9 289 105.00	0.2
Nutrition focused on at‐risk populations	0	$0.00	0	0	$0.00	0.00	0	0	0.0	0	$0.00	0	0	$0.00	0	0	$0.00	0	0	$0.00	0	0	$0.00	0	0	$0.00	0
ARC total	1421	$1 077 668 620.00	100	1268	$559 954 325.00	100	1256	$591 895 243.00	100	1110	$849 559 463.00	100	1127	$580 663 855.00	100	1170	$629 250 267.00	100	1333	$1 078 447 609.00	100	1126	$676 011 044.00	100	9811	$6 043 450 426.00	100
NHMRC																											
Nutrition focused	18	$8 313 837.00	1.02	19	$8 312 894.00	1.06	9	$5 315 048	0.6	21	$12 457 048.00	1.5	16	$11 633 554.70	1.33	14	$8 840 081.00	1.13	18	$19 862 922.45	2.15	11	$12 822 598.00	1.68	126	$87 557 983.15	1.3
Nutrition focused on at‐risk populations	2	$1 575 215.00	0.19	4	$2 233 614.00	0.29	2	$1 562 247	0.2	2	$1 061 999.00	0.13	5	$4 225 564.30	0.48	0	$0.00	0	5	$7 852 962.99	0.85	1	$1 169 419.00	0.15	21	$19 681 021.29	0.3
NHMRC total	1257	$811 901 785.00	100	1093	$780 645 195.00	100	1135	$896 140 626	100	1056	$828 821 760.00	100	1103	$877 678 619.25	100	1045	$783 269 192.00	100	857	$923 247 229.38	100	741	$762 465 859.00	100	8287	$6 664 170 265.63	100
ARC and NHMRC																											
Total nutrition focused	21	$8 885 443.00	0.47	20	$8 523 568.00	0.64	11	$6 064 168.00	0.4	25	$13 978 762.00	0.83	16	$11 633 554.70	0.8	17	$9 917 832.00	0.7	20	$20 786 069.45	1.04	14	$17 057 691.00	1.19	144	$96 847 088.15	0.8
Total nutrition focused on at‐risk populations	2	$1 575 215.00	0.08	4	$2 233 614.00	0.17	2	$1 562 247.00	0.1	2	$1 061 999.00	0.06	4	$4 225 564.30	0.29	0	$0.00	0	5	$7 852 962.99	0.39	1	$1 169 419.00	0.08	21	$19 681 021.29	0.2
Total	2678	$1 889 570 405.00	100	2361	$1 340 599 520.00	100	2391	$1 488 035 869.00	100	2166	$1 678 381 223.00	100	2230	$1 458 342 474.25	100	2215	$1 412 519 459.00	100	2190	$2 001 694 838.38	100	1867	$1 438 476 903.00	100	18 098	$12 707 620 691.63	100

Abbreviations: ARC, Australian Research Council; NHMRC, National Health and Medical Research Council.

**FIGURE 1:**
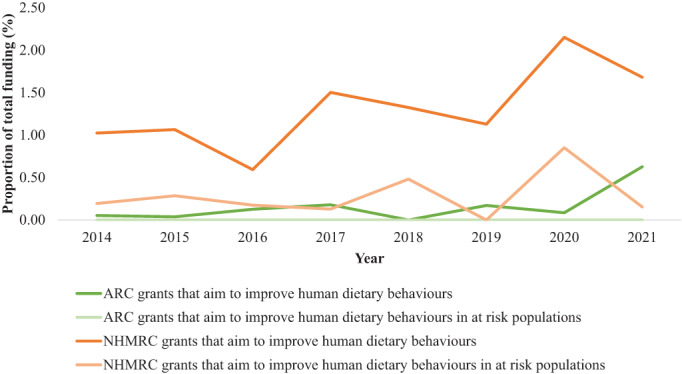
Total proportion of Australian Research Council and National Health and Medical Research Council grant funding by year from 2014 to 2021 that aim to improve human dietary behaviours and in at‐risk populations. ARC, Australian Research Council; NHMRC, National Health and Medical Research Council

Figure 1 shows that there were only minor differences over time. NHMRC and ARC allocated to grants that aim to understand or improve human dietary behaviours increased by 0.66% and 0.58%, respectively, from 2014 to 2021. NHMRC grants that aim to understand or improve human dietary behaviours in at‐risk populations decreased by 0.04% over the 8 years. In 2021, 0.6% of the ARC funding went to grants to understand or improve dietary behaviours, the highest since 2014. Of the NHMRC grants, the year 2020 included the highest percentage of budget allocated to grants to understand or improve human dietary behaviours at 2.15%, with the lowest year being 2016 (0.6%). The average proportion of NHMRC funding allocated to grants that aim to understand or improve dietary behaviours was 1.31% from 2014 to 2021. An average of 0.3% went to grants focussing on nutritional behaviours in at‐risk populations. The total proportion of funding (both ARC and NHMRC) allocated to grants to understand or improve dietary behaviours and dietary behaviours in at‐risk people was 0.75% and 0.15%, respectively, over 2014–2021.

**FIGURE 2:**
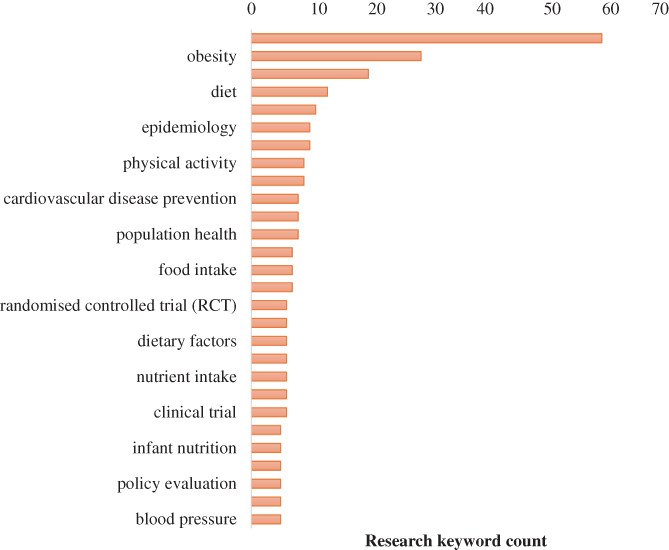
Top research keyword count for National Health and Medical Research Council grants from 2014 to 2021 that aim to improve human dietary behaviours and in at‐risk populations. ARC, Australian Research Council; NHMRC, National Health and Medical Research Council

The top research keywords assigned by applicants to grants (63 total, five per grant for the 126 NHMRC grants) were nutrition (*n* = 60), followed by obesity (*n* = 29), dietary intervention (*n* = 20), diet (*n* = 13), public health (*n* = 11), Aboriginal health (*n* = 10) and epidemiology (*n* = 10; Figure 2). There were a further 464 keywords with four mentions or less.

**FIGURE 3:**
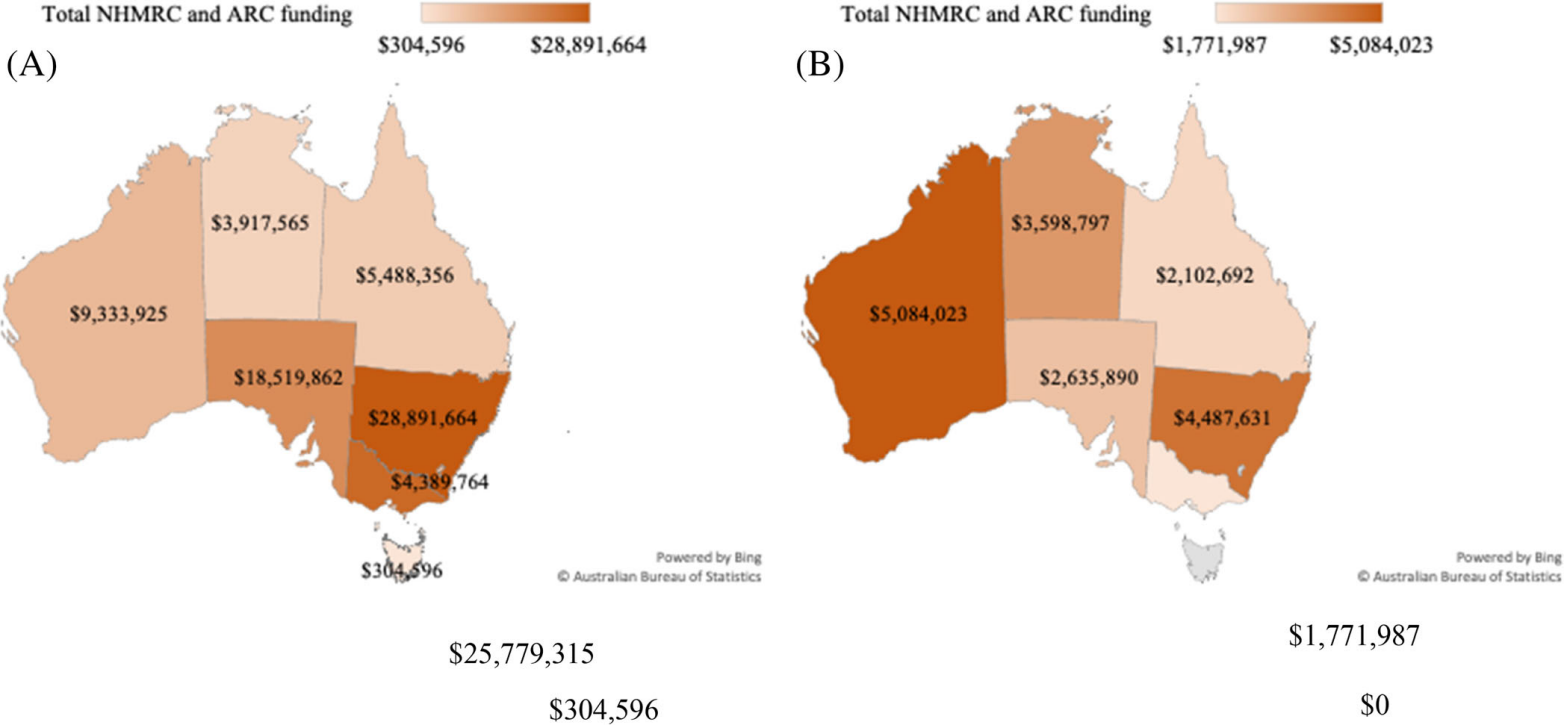
(A) Total funding distribution by state from grants that aim to improve human dietary behaviours from 2014 to 2021 and (B) total funding distribution by state from grants that aim to improve human dietary behaviours in at‐risk populations from 2014 to 2021. ARC, Australian Research Council; NHMRC, National Health and Medical Research Council

Figure 3A outlines the total grants awarded from 2014 to 2021 that aim to understand or improve human dietary behaviours and Figure 3B outlines the total grants that aim to understand or improve human dietary behaviours in at‐risk populations from 2014 to 2021. The figure shows an uneven distribution across the states, with New South Wales researchers receiving the largest funding (total $28 891 664) over the 2014–2021 time frame and the lowest being Tasmania (total $304 596). Investment in nutrition research in at‐risk populations followed a different pattern. Most of the funding was awarded to researchers in Western Australia ($5 084 023), which was more than half (54.4%) of the funding awarded to Western Australia during the time frame. Tasmania received no investment in nutrition research in at‐risk populations from 2014 to 2021.

## 4 DISCUSSION

Overall, from 2014 to 2021, there was minimal NHMRC and ARC funding to grants which primarily focused on understanding or improving dietary behaviours. Although there was more allocation from NHMRC than ARC, the highest mean proportion of funds allocated was 2.15%, with even less going to grants focussing on at‐risk populations such as rural and remote or Aboriginal and Torres Strait Islander peoples, or people living in socioeconomically disadvantaged areas. The very minimal research funding in this area is of particular concern. There appears to be a low investment in improving diets, limiting progress made in addressing dietary risks in Australia, among those most at risk. The lack of investment in nutrition research among at‐risk populations is despite the Federal government's long‐term National Health Plan, which aims to provide equitable health for all, focusing on preventative health, where diet research would play a key role.^30^ There was also an unequal distribution of funding across Australia, with New South Wales receiving most of the funding for research focussed on improving human dietary behaviour. In terms of research focused on at‐risk populations, Western Australian researchers received the most funding. This analysis provides evidence that Australia needs a national and strategic plan for building and streamlining nutrition research. This has been done in other developed countries, including the National Institutes of Health (NIH) in the United States. The NIH has released a strategic plan to accelerate nutrition research from 2020 to 2030, with major focuses including addressing health disparities and nutrition among minority groups.^31^

Our findings appear consistent with the research literature, especially around under‐researched, at‐risk populations. For example, a recent review by Alston and Partridge found that there has been minimal research over the past 20 years, describing interventions to improve dietary behaviours in rural Australia and even less focused on Indigenous Australians.^17^ Another study also found that only a few nutrition interventions aim to improve food supply or food environments in non‐metropolitan areas, in Australia and across the globe.^32^ Our findings are also consistent with a review of NHMRC funding targeting all rural health research (awarded from 2000 to 2014), which highlighted a significant lack of investment relative to the health needs in rural communities.^33^ Our findings support the assumption that rural health research focusing on improving dietary behaviour is still under‐investigated. Limited change in investment has been observed over the 20‐year period between our current study and the deficits in research funding previously highlighted.^33^

Populations of low socioeconomic status in developed countries like Australia tend to have an average of 2.1 years lower life expectancy due to their socioeconomic status alone.^34^ Achieving a healthy diet is further challenged by the perceived and actual cost of healthy food.^35^ Consistent with our findings of low investment in nutrition research aimed at disadvantaged groups, a review by Lewis et al. identified only a few studies investigating the cost of healthy food in Australian populations experiencing high levels of disadvantage.^36^ Additional evidence shows that low socioeconomic groups are not well represented in nationally representative samples that examine patterns in dietary intakes to inform public health initiatives. This therefore is a further limitation to developing new knowledge to address inequities.^37^ The same applies to people with a disability in Australia, another priority population for nutrition research. This population is at an increased risk of poor diet than those without a disability and are at a higher risk of chronic disease.^23^ For example, in 2018, 47% of people living with a disability reported not meeting fruit and vegetable intakes, compared to 41% of those without a disability in Australia.^23^ Despite more than 4.4 million people with a disability in Australia, these data show that no funding has been allocated to research aimed at improving dietary behaviours in this population.^23^

Although it may reflect the position of nutrition‐focused research teams across Australia and the Eastern positioning of the major eight Australian Universities (or ‘Group of Eight’),^38^ total funding distribution was not equal across the states. It is also difficult to ascertain whether research funding in these states was national or multistate in focus or included researchers collaborating across different areas. Nutrition researchers in NSW received the largest total funding across the time frame, perhaps a reflection of being the most populous state in Australia (including the largest Indigenous population in the country) and NSW has the most Universities out of any state.^29^ Due to the higher population numbers, NSW may potentially have the higher nutrition research capacity and capability than other states.^39^ Nutrition research is likely to be limited in this region. Tasmania received the least funding of all the states analysed, and further exploration is needed to understand why existing nutrition research capacity and capability are limited in this state.

Adequate research funding for advancing understanding or improving human dietary behaviours in Australia is critical to developing and translating effective interventions that will enhance nutrition and wellbeing, and prevent non‐communicable diseases.^2^ As such, a lack of funding support for such projects poses a considerable risk to the current and future health of the Australian population. Low investment in translational research has been reported by Zurynski et al., who found most medical research funding in Australia continues to be spent on basic research rather than on health services and public health research. Arguably, translational research in health services and public health research is where much of the translation needs to be embedded to effect change in population diets.^40^ Furthermore, a lack of investment in researching nutritionally at‐risk populations generates an environment where observed health disparities will continue to exist and likely widen, especially for morbidity and mortality associated with dietary risks in these groups.^2^, ^4^, ^23^, ^29^ There are already gaps in the data on dietary intake for at‐risk populations in Australia, even among nationally representative surveys.^24^, ^41^

To address the lack of funding for nutrition research, a national nutrition policy should be considered to guide and direct the prioritisation of diet‐related research into critical areas of need by the nationally competitive funding bodies, including the NHMRC and ARC.^42^ Australia has not had a national nutrition policy for nearly 30 years, despite poor diet being a significant contributor to the estimated $8.6 billion (in 2014–2015) dollars in annual healthcare costs and lost productivity from overweight and obesity in Australia.^43^ Individual dietary risk factors alone have been estimated to cost Australia approximately $561 million annually in productivity impacts.^44^ Given the economic and societal burden of poor nutrition, there have been increasing calls from expert nutrition researchers through the decadal plan for the science of nutrition to develop ‘cost‐effective, equitable population health initiatives developed from accurate knowledge of current diet–health relationships and addressing societal and commercial factors’.^20^ Further research investment in robust nutrition interventions along with knowledge generation studies that include assessments of the drivers of the challenges experienced by at‐risk groups, aligned with a national nutrition policy would significantly enhance this call to action. In addition, a focus on building capacity in food and nutrition research science as whole needs to be considered as a priority by universities, along with creating research coalitions focusing on improving diet in ‘at‐risk’ populations. This will increase the likelihood of greater success of receiving funding.

This study utilised a comprehensive data set describing all ARC and NHRMC grant funding allocated over an 8‐year time frame and used a robust method to categorise and define grants based on search terms and cross‐checks by four experienced researchers. Limitations of this study include that the funding analysed does not include other funding sources, such as government or other foundation funding (such as the National Heart Foundation and Medical Research Futures Fund) and does not consider unfunded nutrition research that may have met the defining criteria, such as that being undertaken by higher degree research students. However, as NHMRC and ARC present as two of the most prestigious categories of funding considered in the Australian academic setting, these results provide an adequate picture for future consideration. It is important to note that the ARC grant scheme does have a lower proportion of funding due to different eligibility criteria compared to NHMRC (i.e. do not support ‘research with direct medical and or human health aims or purpose’), which may also explain the lower funding allocations found for this scheme.^45^ In addition, data from 2015 did not include media summaries, however, search terms and the researcher project summaries were still searched, so there is not expected to be variation in results due to this limitation. It is recognised that some grants may have addressed at‐risk groups but did not mention such groups in the media or plain text summaries, titles or research keywords. However, it is assumed if at‐risk population groups are the critical priority population of the grant proposals, such groups should be mentioned, particularly in the research keywords. Further, we were not able to analyse the allocation of grants based on gender. This could be a significant influence as, broadly, the nutrition field is largely female‐dominated and it has been documented that female researchers have experienced inequity in research funding.^46^ This gender bias could be a factor impacting on grant allocation for nutrition research among at‐risk populations.

Despite the immense potential for optimising diets to improve health in Australia, the NHMRC and the ARC funding for dietary research has been lacking for most of the past decade. Even more concerning is the low investment in research focusing on improving diets in Aboriginal and Torres Strait Islander people, rural, regional and remote residents, people experiencing socioeconomic disadvantage, and no funding among people with disabilities. Inadequate investment in research in these groups presents a real risk for widening health disparities now and into the future. Consideration of these deficiencies is needed when prioritising and designing nationally competitive funding schemes.

## CONFLICT OF INTEREST

The authors declare no conflicts of interest relevant to this study.

## AUTHOR CONTRIBUTIONS

LA and SRP conceived the research idea. LA, RR, SSJ and SRP all coded, analysed, contributed, wrote and reviewed the manuscript.

## Data Availability

The data is publicly available.
